# Supplementary material for: False discovery rates: a new deal
Source: Biostatistics. 2016 Oct 17;18(2):275–94. doi: 10.1093/biostatistics/kxw041 (PMC5379932; doi:10.1093/biostatistics/kxw041)
Supplement: Supplementary Data [file kxw041_Supp.pdf]

Supplementary Information for “False Discovery Rates, A New Deal”

Matthew Stephens

S.1 Model Embellishment Details

This section details some of the model embellishments only briefly mentioned in the paper.

More flexible unimodal distributions

The mixture of normals (2.3) implies that g is not only unimodal, but also symmetric. Furthermore, even some symmetric unimodal distributions, such as those with a flat top, cannot be well approximated by a mixture of zero-centered normals. Therefore, we implemented a more general approach based on

$$g(\cdot;\pi) = \sum_{k=0}^{K} \pi_k f_k(\cdot), \tag{S.1.1}$$

where f_0 is a point mass on 0, and f_k $(k = 1, \dots, K)$ are pre-specified component distributions with one of the following forms:

(i) $f_k(\cdot) = N(\cdot; 0, \sigma_k^2)$, (“ash.n”)

(ii) $f_k(\cdot) = U[\cdot; -a_k, a_k]$, (“ash.u”)

(iii) $f_k(\cdot) = U[\cdot; -a_k, 0]$ and/or $U[\cdot; 0, a_k]$, (“ash.hu”)

where $U[\cdot; a, b]$ denotes the density of a uniform distribution on $[a, b]$. (In (iii) we include both components in the mixture (S.1.1), so a grid of values $a_1, \dots, a_K$ defines $2K + 1$ mixture component densities, and π is a $2K + 1$ vector that sums to 1.)

Moving from (i) to (iii) the representation (S.1.1) becomes increasingly flexible. Indeed, using a large dense grid of σ_k^2 or a_k, (i)-(iii) can respectively approximate, with arbitrary accuracy, (i) any scale mixture of normals; (ii) any symmetric unimodal distribution about 0; (iii) any unimodal distribution about 0. The latter two claims are related to characterizations of unimodal distributions due to Khintchine (1938) and Shepp (1962); see Feller (1971), p158. In other words, (ii) and (iii) provide fully non-parametric estimation for g under the constraints that it is (ii) both unimodal and symmetric, or (iii) unimodal only.

Replace normal likelihood with t likelihood

We generalize the normal likelihood (2.4) by replacing it with a t likelihood:

$$\hat{\beta}_j \,|\, \beta_j, \hat{s}_j \sim T_\nu(\beta_j, \hat{s}_j) \tag{S.1.2}$$

where $T_\nu(\beta_j, \hat{s}_j)$ denotes the distribution of $\beta_j + \hat{s}_j T_\nu$ where T_ν has a standard t distribution on ν degrees of freedom, and ν denotes the degrees of freedom used to estimate $\hat{s}_j$ (assumed known, and for simplicity assumed to be the same for each j). The normal approximation (2.4) corresponds to the limit $\nu \to \infty$. This generalization does not complicate inference when the mixture components f_k in (S.1.1) are uniforms; when the f_k are normal the computations with a t likelihood are considerably more difficult and not implemented.

Equation (S.1.2) is, of course, motivated by the standard asymptotic result

$$(\hat{\beta}_j - \beta_j)/\hat{s}_j \sim T_\nu. \tag{S.1.3}$$

However (S.1.3) does not imply (S.1.2), because in (S.1.3) $\hat{s}_j$ is random whereas in (S.1.2) it is conditioned on. In principle it would be preferable, for a number of reasons, to model the randomness in $\hat{s}_j$; we are currently pursuing this improved approach in joint work with M.Lu.

Non-zero mode

An addition to our software implementation, due to C.Dai, allows the mode to be estimated from the data by maximum likelihood. This involves a simple grid search.

S.2 Implementation Details

Likelihood for π

We define the likelihood for π to be the probability of the observed data $\hat{\beta}$ conditional on $\hat{s}$:

$$L(\pi) := p(\hat{\beta}|\hat{s}, \pi) = \prod_j p(\hat{\beta}_j|\hat{s}, \pi), \tag{S.2.1}$$

where the right hand side comes from our conditional independence assumptions. [One might prefer to define the likelihood as $p(\hat{\beta}, \hat{s}|\pi) = p(\hat{\beta}|\hat{s}, \pi)p(\hat{s}|\pi)$, in which case our definition comes down to assuming that the term $p(\hat{s}|\pi)$ does not depend on π.]

Using the prior $\beta_j \sim \sum_{k=0}^{K} \pi_k f_k(\beta_j)$ given by (S.1.1), and the normal likelihood (2.4), integrating over β_j yields

$$p(\hat{\beta}_j|\hat{s}, \pi) = \sum_{k=0}^{K} \pi_k \tilde{f}_k(\hat{\beta}_j) \tag{S.2.2}$$

where

$$\tilde{f}_k(\hat{\beta}_j) := \int f_k(\beta_j) N(\hat{\beta}_j; \beta_j, \hat{s}_j^2)\, d\beta_j \tag{S.2.3}$$

denotes the convolution of f_k with a normal density. These convolutions are straightforward to evaluate whether f_k is a normal or uniform density. Specifically,

$$\tilde{f}_k(\hat{\beta}_j) = \begin{cases} N(\hat{\beta}_j; 0, \hat{s}_j^2 + \sigma_k^2) & \text{if } f_k(\cdot) = N(\cdot; 0, \sigma_k^2), \\ \frac{\Psi((\hat{\beta}_j - a_k)/\hat{s}_j) - \Psi((\hat{\beta}_j - b_k)/\hat{s}_j)}{b_k - a_k} & \text{if } f_k(\cdot) = U(\cdot; a_k, b_k), \end{cases} \tag{S.2.4}$$

where Ψ denotes the cumulative distribution function (c.d.f.) of the standard normal distribution. If we replace the normal likelihood with the t_ν likelihood (S.1.2) then the convolution for f_k uniform the convolution is still given by (S.2.4) but with Ψ the c.d.f. of the t_ν distribution function. (The convolution for f_k normal is tricky and we have not implemented it.)

Penalty term on π

To make *lfdr* and *lfsr* estimates from our method "conservative" we add a penalty term $log(h(\pi; \lambda))$ to the log-likelihood $\log L(\pi)$ to encourage over-estimation of π_0:

$$h(\pi; \lambda) = \prod_{k=0}^{K} \pi_k^{\lambda_k - 1} \tag{S.2.5}$$

where $\lambda_k \geq 1 \, \forall k$. The default is $\lambda_0 = 10$ and $\lambda_k = 1$, which yielded consistently conservative estimation of π_0 in our simulations (Figure 2b).

Although this penalty is based on a Dirichlet density, we do not interpret this as a "prior distribution" for π: we chose it to provide conservative estimates of π_0 rather than to represent prior belief.

Problems with removing the penalty term in the half-uniform case

It is straightforward to remove the penalty term by setting $\lambda_k = 1$ in (S.2.5). We note here an unanticipated problem we came across when using no penalty term in the half-uniform case (i.e. $f_k(\cdot) = U[\cdot; -a_k, 0]$ and/or $U[\cdot; 0, a_k]$ in (S.1.1)): when the data are nearly null, the estimated g converges, as expected and desired, to a distribution where almost all the mass is near 0, but sometimes all this mass is concentrated almost entirely just to one side (left or right) or 0. This can have a very profound effect on the local false sign rate: for example, if all the mass is just to the right of 0 then all observations will be assigned a very high probability of being positive (but very small), and a (misleading) low local false sign rate. For this reason we do not recommend use of the half-uniform with no penalty.

Optimization

With this in place, the penalized log-likelihood for π is given by:

$$\log L(\pi) + \log h(\pi) = \sum_{j=1}^{n} \log(\sum_{k=0}^{K} \pi_k l_{kj}) + \sum_{k=0}^{K} (\lambda_k - 1) \log \pi_k \tag{S.2.6}$$

where the $l_{kj} := \tilde{f}_k(\hat{\beta}_j)$ are known. This is a convex optimization problem, which can be solved very quickly and reliably using interior point (IP) methods. We used the `KWdual` function from

the R package REBayes (Koenker, 2015), which uses Rmosek (Mosek Aps, 2016). We also found a simple EM algorithm (Dempster et al., 1977), accelerated using the elegant R package SQUAREM (Varadhan and Roland, 2008), to provide adequate performance. In our EM implementation we initialized $\pi_k = 1/n$ for $k = 1, \dots, K$, with $\pi_0 = 1 - \pi_1 - \cdots - \pi_K$, and the one-step updates are:

$$w_{kj} = \pi_k l_{kj} / \sum_{k'} \pi_{k'} l_{k'j} \tag{S.2.7}$$

$$n_k = \sum_j w_{kj} + \lambda_k - 1 \quad \text{[E Step]} \tag{S.2.8}$$

$$\pi_k = n_k / \sum_{k'} n_{k'} \quad \text{[M step]}. \tag{S.2.9}$$

One benefit to the EM algorithm is fewer software dependencies. Both EM and IP methods are implemented in the ashr package; results shown here are from the IP method, but graphs from EM are essentially the same. See http://stephenslab.github.io/ash/analysis/checkIP.html and http://stephenslab.github.io/ash/analysis/IPvsEM.html for comparisons.

Conditional distributions

Given $\hat{\pi}$, we compute the conditional distributions

$$p(\beta_j | \hat{\pi}, \hat{\beta}, s) \propto g(\beta_j; \pi) L(\beta_j; \hat{\beta}_j, \hat{s}_j). \tag{S.2.10}$$

Each posterior is a mixture on $K + 1$ components:

$$p(\beta_j | \hat{\pi}, \hat{\beta}, s) = \sum_{k=0}^{K} w_{kj} p_k(\beta_j | \hat{\beta}_j, \hat{s}_j) \tag{S.2.11}$$

where the posterior weights w_{kj} are computed as in (S.2.7) with $\pi = \hat{\pi}$, and the posterior mixture component p_k is the posterior on β_j that would be obtained using prior $f_k(\beta_j)$ and likelihood $L(\beta_j; \hat{\beta}_j, \hat{s}_j)$. All these posterior distributions are easily available. For example, if f_k is uniform and L is t_ν then this is a truncated t distribution. If f_k is normal and L is normal, then this is a normal distribution.

Choice of grid for σ_k, a_k

When f_k is $N(0, \sigma_k)$ we specify our grid by specifying: i) a maximum and minimum value $(\sigma_{\min}, \sigma_{\max})$; ii) a multiplicative factor m to be used in going from one grid-point to the other, so that $\sigma_k = m\sigma_{k-1}$. The multiplicative factor affects the density of the grid; we used $m = \sqrt{2}$ as a default. We chose $\sigma_{\min}$ to be small compared with the measurement precision ($\sigma_{\min} = \min(\hat{s}_j)/10$) and $\sigma_{\max} = 2\sqrt{\max(\hat{\beta}_j^2 - \hat{s}_j^2)}$ based on the idea that $\sigma_{\max}$ should be big enough so that $\sigma_{\max}^2 + \hat{s}_j^2$ should exceed $\hat{\beta}_j^2$. (In rare cases where $\max(\hat{\beta}_j^2 - \hat{s}_j^2)$ is negative we set $\sigma_{\max} = 8\sigma_{\min}$.)

When the mixture components f_k are uniform, we use the same grid for the parameters a_k as for σ_k described above.

Our goal in specifying a grid was to make the limits sufficiently large and small, and the grid sufficiently dense, that results would not change appreciably with a larger or denser grid. For a specific data set one can of course check this by experimenting with the grid, but these defaults usually work well in our experience.

Dependence of effects on standard errors

The model (3.11) for general α can be fitted using the algorithm for $\alpha = 0$. To see this, define $b_j := \beta_j/\hat{s}_j^\alpha$, and $\hat{b}_j := \hat{\beta}_j/\hat{s}_j^\alpha$. Then $\hat{b}_j$ is an estimate of b_j with standard error $\hat{s}'_j := \hat{s}_j^{1-\alpha}$. Applying the algorithm for $\alpha = 0$ to effect estimates $\hat{b}_1, \ldots, \hat{b}_J$ with standard errors $\hat{s}'_1, \ldots, \hat{s}'_J$ yields a posterior distribution $p(b_j|\hat{s}_j, \hat{b}_j, \hat{\pi}, \alpha)$, which induces a posterior distribution on $\beta_j = b_j\hat{s}_j^\alpha$.

S.3 Supplementary Figures and Tables

Scenario	Alternative distribution, g_1
spiky	$0.4N(0, 0.25^2) + 0.2N(0, 0.5^2) + 0.2N(0, 1^2), 0.2N(0, 2^2)$
near normal	$2/3N(0, 1^2) + 1/3N(0, 2^2)$
flattop	$(1/7)[N(-1.5, .5^2) + N(-1, .5^2) + N(-.5, .5^2)+$ $N(0, .5^2) + N(0.5, .5^2) + N(1.0, .5^2) + N(1.5, .5^2)]$
skew	$(1/4)N(-2, 2^2) + (1/4)N(-1, 1.5^2) + (1/3)N(0, 1^2) + (1/6)N(1, 1^2)$
big-normal	$N(0, 4^2)$
bimodal	$0.5N(-2, 1^2) + 0.5N(2, 1^2)$

Table 1: Summary of simulation scenarios considered

References

Dempster, A. P., N. M. Laird, and D. B. Rubin (1977). Maximum likelihood estimation from incomplete data via the EM algorithm (with discussion). *Journal of the Royal Statistical Society, series B 39*, 1–38.

Feller, W. (1971). An introduction to probability and its applications, vol. ii. *Wiley, New York*.

Khintchine, A. Y. (1938). On unimodal distributions. *Izv. Nauchno-Isled. Inst. Mat. Mech. Tomsk. Gos. Univ 2*, 1–7.

Koenker, R. (2015). *REBayes: Empirical Bayes Estimation and Inference in R.* R package version 0.58.

Mosek Aps (2016). *Rmosek: The R to MOSEK Optimization Interface.* R package version 7.1.2.

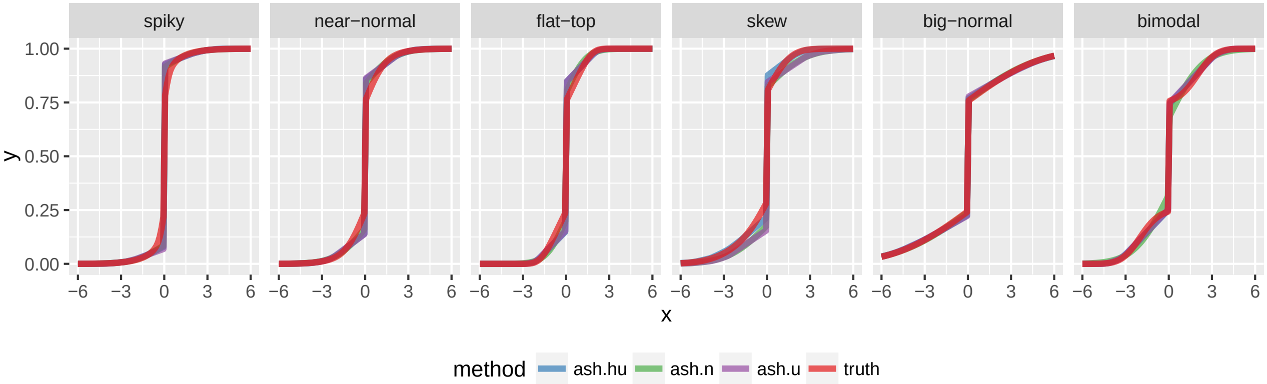

(a) Average estimated cdfs across ~ 10 data sets compared with truth; methods here use penalty (S.2.5) which leads to systematic overestimation of π_0 in some scenarios.

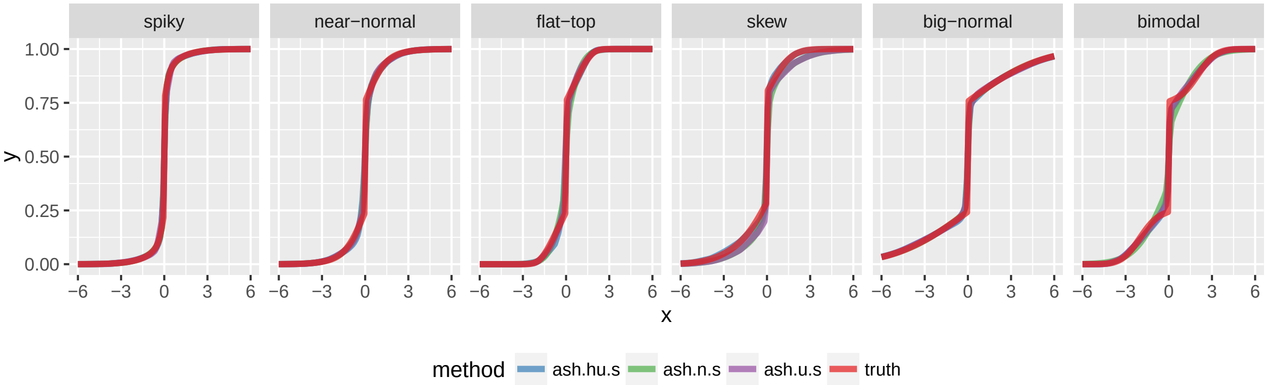

(b) Average estimated cdfs across ~ 10 data sets compared with truth; methods here do not use penalty (S.2.5) so π_0 is not systematically overestimated. Systematic differences from the truth in "skew" and "bimodal" scenarios highlight the effects of model mis-specification.

Figure 1: Comparisons of average estimated cdfs of g with and without penalty term. See Figure 2b for simulation scenarios. In most cases the three different ash methods are very similar and so the lines lie on top of one another.

Shepp, L. (1962). Symmetric random walk. *Transactions of the American Mathematical Society*, 144–153.

Varadhan, R. and C. Roland (2008). Simple and globally convergent methods for accelerating the convergence of any em algorithm. *Scandinavian Journal of Statistics 35*(2), 335–353.

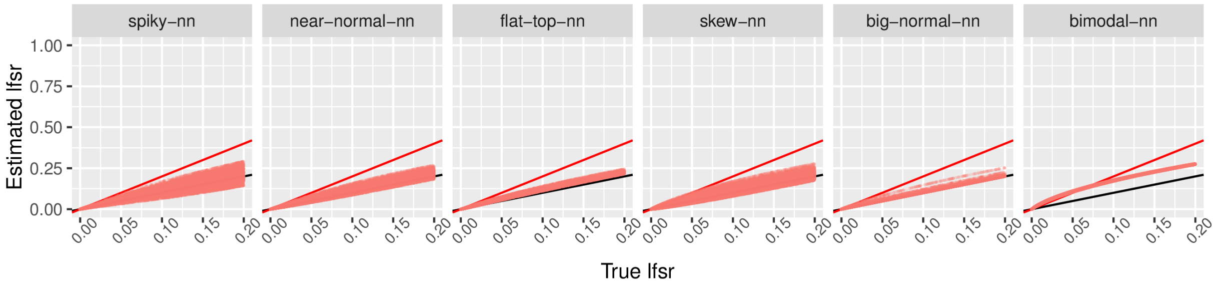

(a) Comparison of true and estimated *lfsr* when data are simulated with no point mass at zero ($\pi_0 = 0$), and also analyzed by `ash` with no point mass on 0 (and mixture of normal components for g). Black line is $y = x$ and red line is $y = 2x$. The results illustrate how estimates of *lfsr* can be more accurate in this case. That is, assuming there is no point mass can be beneficial if that is indeed true.

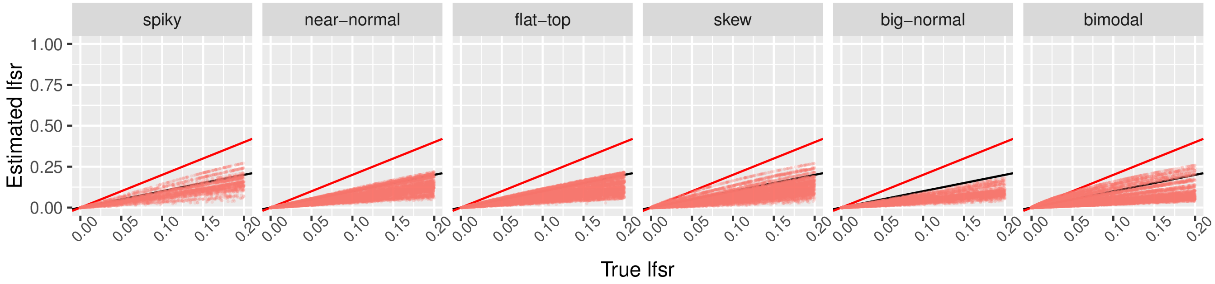

(b) Comparison of true and estimated *lfsr* when data are simulated with point mass at zero (drawn uniformly from [0,1] in each simulation), but analyzed by `ash` with no point mass on 0 (and mixture of normal components for g). Black line is $y = x$ and red line is $y = 2x$. The results illustrate how estimates of *lfsr* can be anti-conservative if we assume there is no point mass when the truth is that there is a point mass.

Figure 2: Illustration of effects of excluding a point mass from the analysis.

	spiky	near-normal	flat-top	skew	big-normal	bimodal
ash.n.s	0.95	0.95	0.95	0.95	0.96	0.96
ash.u.s	0.94	0.95	0.95	0.94	0.96	0.96
ash.hu.s	0.88	0.92	0.92	0.92	0.93	0.93

(a) All observations

	spiky	near-normal	flat-top	skew	big-normal	bimodal
ash.n.s	0.95	0.95	0.98	0.93	0.95	0.97
ash.u.s	0.89	0.92	0.90	0.92	0.94	0.94
ash.hu.s	0.89	0.92	0.91	0.94	0.95	0.94

(b) "Significant" negative discoveries.

	spiky	near-normal	flat-top	skew	big-normal	bimodal
ash.n.s	0.94	0.94	0.92	0.88	0.95	0.94
ash.u.s	0.93	0.93	0.92	0.88	0.95	0.95
ash.hu.s	0.34	0.60	0.52	0.54	0.79	0.82

(c) "Significant" positive discoveries.

Table 2: Table of empirical coverage for nominal 95% lower credible bounds for methods *without* the penalty term).
